# Herbal medicine for cervicogenic dizziness: A protocol for a systematic review and meta-analysis

**DOI:** 10.1097/MD.0000000000023852

**Published:** 2020-12-18

**Authors:** Hyunjoo Oh, Seungwon Shin, Euiju Lee

**Affiliations:** a Department of Clinical Korean Medicine, Graduate School, Kyung Hee University, Seoul, Republic of Korea; b National Agency for Development of Innovative Technologies in Korean Medicine, National Development Institute of Korean Medicine, Seoul, Republic of Korea, Department of Clinical Korean Medicine, Graduate School, Kyung Hee University; c Department of Sasang Constitutional Medicine, College of Korean Medicine, Kyung Hee University, 23 Kyungheedae-ro, Dongdaemun-gu, Seoul, Republic of Korea.

**Keywords:** cervicogenic dizziness, herbal medicine, protocol, systematic review

## Abstract

**Background:**

Herbal medicines are empirically used to treat cervicogenic dizziness. However, till date there have been no systematic review to evaluate the efficacy and safety of these medicines. Therefore, this study protocol describes the methods for evaluating the efficacy and safety of herbal medicine for cervicogenic dizziness.

**Methods and analysis:**

The following electronic academic databases will be searched up to December 2019 without language or publication status restrictions: Medical Literature Analysis and Retrieval System Online (MEDLINE), Excerpta Medica database (EMBASE), and the Cochrane Central Register of Controlled Trials (CENTRAL), together with Korean, Chinese, and Japanese databases. Any randomized controlled trials related to herbal medicine for cervicogenic dizziness will be included. The functional outcomes and the vertebrobasilar artery hemodynamic states will be evaluated as primary outcomes. The total effective rate, hematological conditions, and adverse events will be assessed as secondary outcomes. Study selection, data extraction, quality assessment of studies, and qualitative evaluation of clinical evidence will be performed by 2 independent reviewers. The methodological quality of the included studies will be evaluated using a revised Cochrane risk-of-bias tool for randomized trials. The strength of evidence from the included data will be evaluated using the Grading of Recommendations Assessment, Development, and Evaluation approach. Data synthesis will be performed as either a fixed-effects or a random-effects model using Review Manager software version 5.3. The results will be reported as a risk ratio for dichotomous outcomes and as a mean difference or standardized mean difference for continuous outcomes.

**Ethics and dissemination:**

No ethical approval is required since the individual clinical information of the patient is not used. The findings of this systematic review will be disseminated through the peer-reviewed publications or conference presentations.

**Review Registry Unique Identifying Number:**

reviewregistry1036.

## 1 Introduction

Cervicogenic dizziness (CGD), also known as cervical dizziness or cervical vertigo, is one of the major causes of dizziness.^[1]^ CGD originates from the cervical spine,^[2]^ and it usually results in a variety of coexisting symptoms such as headache, unsteadiness, lightheadedness, perceptions of spinning, nausea, and general disorientation, together with neck pain or stiffness.^[3–5]^ The prevalence of CGD is estimated to 6.4% to 8.5%.^[6–8]^ In particular, 65% to 66% of dizziness in elderly patients is attributed to cervical spine dysfunction.^[9,10]^ It has been reported that the proportion of patients with CGD is gradually increasing in the total number of patients with dizziness.^[11]^ However, the pathogenesis of CGD is unclear. The most common theory is that CGD occurs due to a problem in the proprioceptive system of the neck.^[12]^ When this system is damaged due to muscular fatigue, degeneration, or trauma, nonspecific sensations including dizziness occur due to disharmonic hyperactivity of the cervical mechanoreceptors located in the joints, ligaments, and muscle spindles.^[10,13,14]^ According to a recent study, CGD is classified neural types, which includes degenerative cervical spine disorder, whiplash-associated disorder, and Barre–Lieou syndrome, and vascular types, which includes Bow Hunters syndrome and Beauty Parlour syndrome, based on etiopathological mechanisms. However, these diseases also overlap because they do not have completely distinct mechanisms.^[15]^ Consequently, there are no established diagnostic criteria for CGD, and physicians usually diagnose patients with complain of dizziness and neck pain without other neurological or neuro-otological causes of dizziness as cervicogenic.^[16,17]^

The treatment of CGD has not been standardized yet; therefore, previous studies have explored a variety of treatments, including physical therapies,^[3,4,8,10,18–22]^ surgery,^[10,16]^ topical drug injection,^[11,23]^ acupuncture therapy,^[24,25]^ and medications, such as muscle relaxants, opioid drugs, nonsteroidal anti-inflammatory drugs, and anxiolytic drugs, in combination with Chinese herbal medicines, to improve the severity and frequency of dizziness, by relaxing muscles and ameliorating abnormal proprioceptive sensibility or impaired blood flow in the cervical region. Among these, herbal medicine is empirically used in combination with other treatments owing to its known therapeutic effect on CGD by suppressing pain and improving blood circulation in the human body.^[24,26]^ However, there has been no systematic verification of the efficacy and safety of herbal medicine for CGD that can support clinical evidence. In this study, we will analyze and evaluate the efficacy and safety of herbal medicine for CGD through a systematic literature review.

## 2 Methods

### 2.1 Study registration

The study protocol for this systematic review has been registered with the Research Registry (Review Registry Unique Identifying Number: reviewregistry1036) on November 19, 2020. There will be no amendments that cause significant distortion in the study design. If certain details in the study protocol are changed, they will be tracked and dated in the Research Registry. This study protocol followed the Preferred Reporting Items for Systematic Review and Meta-Analysis Protocols (PRISMA-P) 2015 statement.^[27]^

### 2.2 Data sources and search strategy

The following 11 electronic academic databases will be searched up to December 2019 without language or publication status restrictions: 4 English databases (Medical Literature Analysis and Retrieval System Online [MEDLINE] via PubMed, Excerpta Medica database [EMBASE] via Elsevier, the Cochrane Central Register of Controlled Trials [CENTRAL], and KoreaMed), 6 Korean databases (Korean Studies Information Service System [KISS], Research Information Sharing Service [RISS], National Digital Science Library [NDSL], Korean Medical Database [KMbase], and Database Periodical Information Academic [DBpia]), 1 Chinese database (China National Knowledge Infrastructure [CNKI]), and 1 Japanese database (Citation Information by NII [CiNii]). In addition, we will perform a manual search on Google Scholar to identify further studies mentioned in the reference lists, if necessary.

As for the search strategies, we will use the search terms consisting of the disease part and the intervention part, tailored to the language and search form of each database. Search strategies have been set up broadly for sensitive searches. For example, the search strategies for MEDLINE are shown in Table 1.

**Table 1:** Search strategies for MEDLINE.

#1 “Dizziness”[MH] OR “Vertigo”[MH] OR “Syncope”[MH] OR “Linkage Disequilibrium”[MH] OR “Dizziness”[TIAB] OR “Orthostasis”[TIAB] OR “Lightheadedness”[TIAB] OR “Light-Headedness”[TIAB] OR “Light Headedness”[TIAB] OR “Vertigos”[TIAB] OR “Vertigo”[TIAB] OR “Spinning”[TIAB] OR “Syncopes”[TIAB] OR “Fainting”[TIAB] OR “Presyncope”[TIAB] OR “Presyncopes”[TIAB] OR “Syncope”[TIAB] OR “Syncopal”[TIAB] OR “Drop Attack”[TIAB] OR “Drop Attacks”[TIAB] OR “Disequilibrium”[TIAB] OR “Disequilibriums”[TIAB] #2 “Plants, Medicinal”[MH] OR “Drugs, Chinese Herbal”[MH] OR “Phytotherapy”[MH] OR “Plant Extracts”[MH] OR “Medicinal plants”[TIAB] OR “Medicinal Plant”[TIAB] OR “plant extracts”[TIAB] OR Herbs[TIAB] OR Herb[TIAB] OR Herbal[TIAB] OR “phytotherapy”[TIAB] #3 (traditional[TIAB] OR Chinese[TIAB]) AND (medicine[TIAB] OR drugs[TIAB] OR drug[TIAB]) #4 “Mahwangbujaseshin-tang”[TIAB] OR “Sopung-san”[TIAB] OR “Dodam-tang”[TIAB] OR “Woohwangchungshim-won”[TIAB] OR “Sahyangsohap-won”[TIAB] OR “Jinmu-tang”[TIAB] OR “Gongjin-dan”[TIAB] OR “Jeungikgwiyong-won”[TIAB] OR “Yeonjueum”[TIAB] OR “Jaeumyeongsin-tang”[TIAB] OR “Hyeongbangjiwhang-tang”[TIAB] OR “Cheonghunhwadam-tang”[TIAB] OR “Woohwangchungshim-won”[TIAB] OR “Yukwooltnag”[TIAB] OR “Yukwool-tang”[TIAB] OR “Daejo-hwan”[TIAB] OR “Cheonmabanhwa-Tang”[TIAB] OR “Banhabaekchulchoenma-tang”[TIAB] OR “Chilgi-tang”[TIAB] OR “Hyeongbangdojok-san”[TIAB] OR “Younggaechulgam-tang”[TIAB] OR “Pyungwi-san”[TIAB] OR “Zizyphi Spinosi Semen”[TIAB] OR “Buzhongyiqi-Tang”[TIAB] OR “Hyeongbangsabaek-san”[TIAB] OR “Dojeokgamgi-tang”[TIAB] OR “Gihwangbaekho-tang”[TIAB] OR “Jaeumkunbi-tang”[TIAB] OR “Daeshiho-tang”[TIAB] OR “Bunsimgi-eum”[TIAB] OR “Hyunggaeyungyo-tang”[TIAB] OR “Gwakhyangjunggi-san”[TIAB] OR “Sunghyangchungi-san”[TIAB] OR “Hyangsayangwi-tang”[TIAB] OR “Palmulgunja-tang”[TIAB] OR “Bosimgunbi-tang”[TIAB] OR “Chengsimyeonja-tang”[TIAB] OR “Hyangsayukgunja-Tang”[TIAB] OR “Goepoong-san”[TIAB] OR “Younggaechulgam-tang”[TIAB] OR “Jaeumgeonbi-tang”[TIAB] OR “Sagunja-tang”[TIAB] OR “Yijin-tang”[TIAB] OR “Leejung-tang”[TIAB] OR “Taeeumjowui-tang”[TIAB] OR “Daekumeumja”[TIAB] OR “yookmijihwang-tang”[TIAB] OR “Bangpungtongsung-san”[TIAB] OR “Yanghyeolgeopung-tang”[TIAB] OR “Samul-tang”[TIAB] OR “Soonkiwhalwheul-tang”[TIAB] OR “Yupoongyangyeong-tang”[TIAB] OR “Gamisachi-tang”[TIAB] OR “Hyangsapyeongwi-san”[TIAB] OR “Yangkyuksanwha-tang”[TIAB] OR “Taeksa-tang”[TIAB] OR “Zexie-tang”[TIAB] OR “Joganiknoe-tang”[TIAB] OR “Samhwangsasim-tang”[TIAB] OR “Yanghyeolgeopung-tang”[TIAB] OR “yangxuequfeng-tang”[TIAB] OR “Yanghyeolgeopung-tang”[TIAB] OR “yangxuequfeng-tang”[TIAB] OR “Choweseuncheng-tang”[TIAB] OR “GamiJihwangyeumja”[TIAB] OR “Sibimijihwang-tang”[TIAB] OR “Mihuedungsikjang-tang”[TIAB] OR “Kamiguibitang”[TIAB] OR “Dodamhwalhultang”[TIAB] OR “Kamiguibi-tang”[TIAB] OR “Dodamhwalhul-tang”[TIAB] OR “Shin-Ki-Hwan”[TIAB] OR “Jengjengamiygin-tang”[TIAB] OR “Arrowroot Puerariae Radix”[TIAB] OR “Salvia miltiorrhiza Bunge”[TIAB] OR “Chrysanthemum indicum L.”[TIAB] OR “Vitex rotundifolia Seeds”[TIAB] OR “Rehmanniae Radix Preparata”[TIAB] OR “Lycii fructus”[TIAB] OR “Disocorea batatas”[TIAB] OR “Injinoryeong-san”[TIAB] OR “Cheonginigeuk-Tang”[TIAB] OR “Melonis Calyx”[TIAB] OR “Yukmijihwangwon”[TIAB] OR “Bosimsahwacheonggan-tang”[TIAB] OR “Hachulbosim-tang”[TIAB] OR “Yukmijihwang-won”[TIAB] OR “Hwaryongibcheoneum”[TIAB] OR “Yeongsindodam-tang”[TIAB] OR “Cheongsinhaeo-tang”[TIAB] OR “Sangcheongbaekbuja-hwan”[TIAB] OR “Junghyeon-tang”[TIAB] OR “Sihogayongmo-tang”[TIAB] OR “Ikgichongmyeong-tang”[TIAB] OR “chongi-tang”[TIAB] OR “Jeongansikpung-tang”[TIAB] #5 #2 OR #3 OR #4 #6 #1 AND #5

### 2.3 Eligibility criteria

#### 2.3.1 Types of studies

All randomized controlled trials related to our subject will be included. Other designs, such as pre-clinical/animal studies, case reports, retrospective studies, literature research, qualitative research, review studies, and conference presentations will be excluded.

#### 2.3.2 Participants

All CGD patients treated with herbal medicine will be considered as subjects of our study, with no restrictions on ethnicity, nationality, sex, age, or biological status.

#### 2.3.3 Interventions and comparisons

Herbal medicine with any formulation administered orally, such as decoction, capsules, tablets, pills, and powders, will be considered as experimental interventions. There will be no limitation on the combination or number of herbs, dosage of medicine, frequency, or duration of treatment. However, studies will be excluded if they have insufficient information on the composition of the herbal medicine. Meanwhile, no treatment, placebo, or other conventional medical treatments will be considered as control interventions; however, comparisons between different combinations of herbal medicines will be excluded. In addition, studies involving herbal medicine combined with other treatments as experimental interventions will be included under the condition of equal application of the treatment except herbal medicine in both the experimental group and the control group.

#### 2.3.4 Outcomes

The primary outcomes are as follows:

1. Functional outcomes measured by validated scales, encompassing those used to simply assess the degree of CGD symptoms (e.g., numeric rating scale) to those used to evaluate overall functions of CGD patients (e.g., Functional Scale for Cervical spondylosis of the vertebral artery type)
2. Vertebrobasilar artery hemodynamic states (e.g., average velocity of blood flow).

The secondary outcomes are as follows:

1. The total effective rate
2. Hematological conditions (e.g., levels of serum fibrinogen, plasma endothelin, and calcitonin gene-related peptide)
3. Adverse events

### 2.4 Study selection

**Figure 1:**
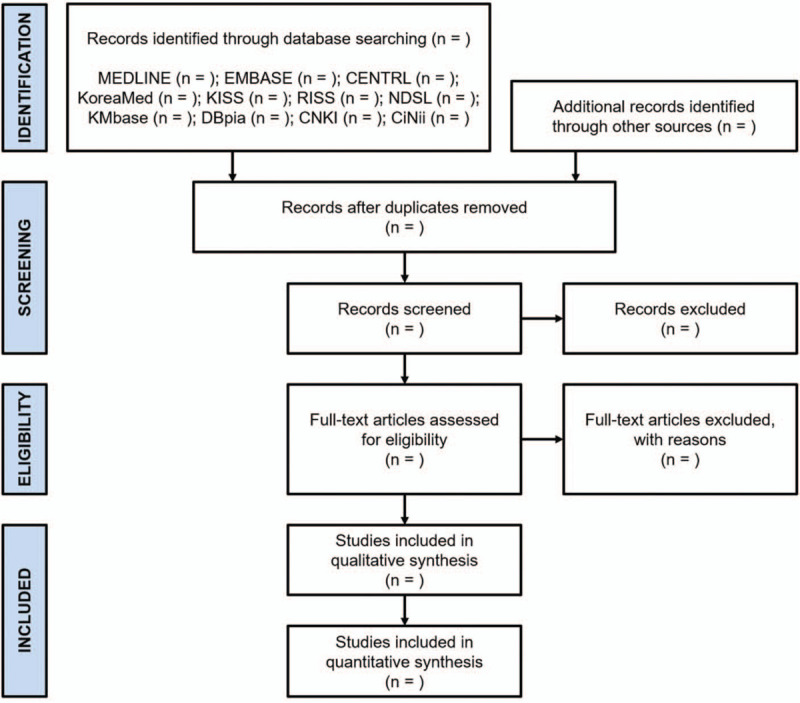
PRISMA flow diagram of the literature search for cervicogenic dizziness and herbal medicine. CENTRAL = Cochrane Central Register of Controlled Trials, CiNii = Citation Information by NII, CNKI = China National Knowledge Infrastructure, DBpia = Database Periodical Information Academic, KISS = Korean Studies Information Service System, KMbase = Korean Medical Database, NDSL = National Digital Science Library, RISS = Research Information Sharing Service.

Two independent reviewers (HO and SS), blinded to each other, will screen and assess the eligibility of all retrieved studies based on the aforementioned criteria. For studies identified through the database search or other sources, duplicates will be removed, titles and abstracts of the remaining studies will be screened, and their eligibility will be sequentially evaluated by full-text review. Any divergence in agreement will be resolved through discussion with other researchers at each step of the study selection process. The process will be reported following the PRISMA statement (Fig. 1).^[28]^

### 2.5 Data extraction

Data from the included studies will be extracted by 2 independent reviewers (HO and SS) using a predefined data acquisition form. This form will include 4 main domains: general information (title, authors, country of study, journal, and year of publication), participant characteristics (age, sex, and diagnostic criteria), details of intervention and comparison (number of participants in each group, formulation and name of herbal medicine, number of herbs, dosage of medicine, comparison, frequency or duration of the treatment, and follow-up information), and outcomes (primary and secondary outcomes and adverse events). The data on herbal medicine will be extracted according to the Consolidated Standards of Reporting Trials Extension for Chinese Herbal Medicine Formulas 2017.^[29]^ The discrepancy will be identified and resolved through discussion with other researchers. Missing or insufficient data will be requested from the corresponding authors of the relevant studies.

### 2.6 Quality assessment

The methodological quality of the included studies will be evaluated using a revised Cochrane risk-of-bias tool for randomized trials (RoB 2).^[30]^ Bias domain for risk-of-bias assessment will include the following 5 domains:

1. bias arising from the randomization process,
2. bias due to deviations from intended interventions,
3. bias due to missing outcome data,
4. bias in the measurement of the outcome, and
5. bias in the selection of the reported result.

The risk of bias will be evaluated as “low-risk,” “high-risk,” or “some concerns” by the 2 reviewers independently, and any divergence in agreement will be resolved through discussion with the third reviewer if necessary. All studies evaluated as “low risk” in all domains will be defined as high quality. The result of risk of bias assessment will be presented using Review Manager (RevMan) software version 5.3 (Cochrane Collaboration, Oxford, UK).

Moreover, the strength of evidence from the included data will be evaluated using the Grading of Recommendations Assessment, Development, and Evaluation (GRADE) approach.^[31]^ The following items will be assessed: risk of bias, inconsistency, indirectness and imprecision of the results, and publication bias. The quality of the body of evidence will be evaluated as “high,” “moderate,” “low,” or “very low,” and the result will be presented in a “Summary of findings” table.

### 2.7 Data synthesis

Data on the efficacy of herbal medicine for CGD will be analyzed using RevMan software version 5.3. A quantitative synthesis (meta-analysis) will be performed if the included studies are sufficiently homogenous. A subgroup analysis will be conducted by considering different types of comparison such as no treatment, placebo, and other conventional medical treatment, if possible. The results will be reported as a risk ratio for the dichotomous outcome and as a mean difference or standardized mean difference for the continuous outcome, together with 95% confidence intervals.

Statistical heterogeneity among studies will be assessed by computing *I*^2^ statistics. The data will be pooled using a random-effects model if included studies have significant heterogeneity (considered *I*^2^ values ≥50% as “Substantial heterogeneity” and ≥75% as “Considerable heterogeneity,” and also considered both as “significant”). Otherwise, a fixed-effects model will be applied.^[32]^ Sensitivity analyses will be performed to increase the robustness of results by excluding studies with high risks of bias and outliers. Funnel plots will be used to detect publication bias if the number of studies is sufficient. Meanwhile, data on the safety of herbal medicine for CGD will be described qualitatively.

### 2.8 Ethics and dissemination

No ethical approval is required since the patients individual clinical information is not used. The findings of this systematic review will be disseminated through the peer-reviewed publications or conference presentations.

## 3 Discussion

It is necessary to develop strategies that are effective in treating and managing CGD, the incidence of which is increasing gradually, since the majority of the patients with dizziness are the elderly, and CGD accounts for an enormous proportion of dizziness in the elderly.^[9,10]^ Herbal medicine is a treatment that has been used empirically for CGD patients, sometimes combined with other treatments. Some clinical evidence has suggested that herbal medicine is effective in relieving symptoms of CGD and promoting cervical blood circulation as reported by previous studies.^[24,26]^ However, there has been no systematic review to evaluate the efficacy and safety of herbal medicine for CGD. This research will help clinicians prescribe herbal medications for CGD patients based on systematic clinical evidence and provide ideas for further clinical trials; additionally, it will expand our knowledge of potential medications for CGD treatment.

## Author contributions

**Conceptualization:** Hyunjoo Oh.

**Funding acquisition:** Hyunjoo Oh, Euiju Lee.

**Methodology:** Hyunjoo Oh, Seungwon Shin.

**Supervision:** Euiju Lee.

**Writing – original draft:** Hyunjoo Oh.

**Writing – review & editing:** Seungwon Shin, Euiju Lee.
